# Loss of chance associated with sub-optimal HPV vaccination coverage rate in France

**DOI:** 10.1016/j.pvr.2017.02.004

**Published:** 2017-02-22

**Authors:** Mathieu Uhart, Marjorie Adam, André Dahlab, Xavier Bresse

**Affiliations:** Sanofi Pasteur MSD, 162 avenue Jean Jaures, 69367 Lyon, France

**Keywords:** Human papillomavirus, HPV vaccine, Vaccine coverage rate, France, Public health

## Abstract

**Introduction:**

Since 2007, HPV vaccination programs have been implemented in Europe. Significant real-life impact has already been reported in countries where the programs have been successfully implemented. In France, HPV vaccination coverage rate (VCR) is currently one of the lowest in Europe. This represents a missed opportunity for individuals who will not be protected. The study aimed to estimate the consequences of the sub-optimal VCR.

**Methods:**

A dynamic transmission model was calibrated to the French setting. Outcomes resulting from the vaccination of girls with quadrivalent HPV vaccine according to two theoretical VCR: 17% and 70%, reflecting the range of VCRs in Western Europe, were evaluated.

**Results:**

Over 100 years, with the current low VCR, an additional 85,000 cancers, 28,000 deaths and more than 5 million avertable disease events overall would occur compared with a 70% VCR. At steady state, the 17% VCR was estimated to be associated with an additional 1700 cancers, 600 deaths and 66,000 avertable disease events each year, compared with a ‘standard’ EU VCR.

**Conclusion:**

The loss of chance associated with sub-optimal VCR is substantial for the French population and could amount to the occurrence of hundreds of avoidable deaths and thousands of disease events annually.

## Highlights

• HPV vaccination coverage rate in France is suboptimal.
• Loss of chance associated with the sub-optimal VCR is tremendous for the French population.
• Communicating on the magnitude of the loss of opportunity of not vaccinating the population is important.

## 1 Introduction

Human papillomaviruses (HPVs) are responsible for cancers in both genders at different anatomical sites, including cervix uteri, vulva, vagina, anus, penis and oropharynx [1], as well as other diseases, such as anogenital warts and recurrent respiratory papillomatosis [2]. In France, almost 4700 new cases of cancer of the cervix, vulva, vagina and anus, attributable to HPV, are estimated to occur every year [3]. Cervical cancer is the fourth most common cancer in women under the age of 45, killing more than 1000 women each year in France and is the most well-known HPV cancer [4]. Almost 100 HPV genotypes have been identified, although they are not all ‘high-risk’ or oncogenic genotypes. It has been estimated that about 70% of cervical cancers are due to two HPV genotypes, HPV16 and HPV18, and 90% of anogenital warts are caused by HPV6 and HPV11 [3].

Early detection of cervical precancerous lesions through organized screening programs has helped to lower the incidence rates of cervical cancer but the rate of decline has slowed down since the beginning of the 21st century. In France, screening is mostly opportunistic, with women aged between 25 and 65 years undergoing a Pap test every 3 years (after 2 normal Pap tests one year apart) [5]. Unlike for cervical cancer in women, no systematic and effective screening exists for other HPV-related cancers.

Since 2007, HPV vaccination programs have been implemented in most European countries for the prevention of cervical cancer, targeting girls aged between 9 and 14 years, depending on the local recommendations. Some countries, like France, have also implemented catch-up vaccination for girls aged between 15 and 19. Early and significant real-world impact of HPV vaccination has been reported in countries were the vaccination programs have been successfully implemented [6]. In Australia, where broad and high vaccine coverage was rapidly achieved with the quadrivalent HPV vaccination, containing HPV6/11/16/18, the reduction in high-grade cervical precancerous lesions reached 54% after 7 years and genital warts were almost eliminated among young women [7], [8], [9], [10]. Similar results have been observed in other countries like in Sweden and Denmark [11], [12], [13] after the successful implementation of quadrivalent HPV vaccination programs. In England and Scotland, where an initial national HPV immunization program was implemented with the bivalent HPV vaccine containing HPV16/18, a decline in the prevalence of vaccine and some non-vaccine HPV types and a reduction of low- and high-grade CIN was associated with high vaccine uptake.

Although vaccination coverage rates are >80% in the United Kingdom and Portugal, coverage rates remain sub-optimal in many European countries, including France [14], where HPV vaccination coverage is the lowest, with a cumulative coverage rate in 2015 of 17.2% in girls aged 16 years [15]. The differences in the implementation of HPV vaccination programs and coverage rates may lead to a two-tiered Europe, which could hamper the control of HPV-associated cancers in Europe and contribute to health inequality in the region. This represents a missed opportunity for thousands of individuals who will not be protected against vaccine-preventable cancers and diseases.

Many publications have analyzed the determinants of vaccination uptake [16], [17], [18], [19], [20], but only a few have analyzed the consequences of sub-optimal vaccine coverage. The public health impact of unsatisfactory vaccination coverage rates in France has been estimated for several vaccines, but not for HPV [21]. The objective of the present study was therefore to estimate the public health consequences of the low HPV vaccination coverage rate in France and quantify the associated loss of chance.

## 2 Materials and methods

### 2.1 Mathematical dynamic transmission model

A previously published US model, simulating the natural history of HPV-infections and related diseases, caused by genotypes 6, 11, 16, 18, was adapted to France to estimate the public health impact of the quadrivalent HPV vaccine [22], [23], [24], [25]. More details about the model can be found in the Supplementary material and in a previous publication [24].

The model has three connected modules:

(1) a demographic model that defines the demographic characteristics of the population and describes how persons enter, transition and exit the model;
(2) an epidemiologic module that simulates HPV transmission and the occurrence of HPV-related diseases;
(3) an economic model that estimates the costs and quality of life associated with the screening, vaccination and management of the disease

The epidemiologic module consists of 14 separate and independent models to take into account the different HPV genotypes and diseases. The model accounts for the transmission dynamics of four HPV genotypes: 16, 18, 6, 11, and simulates the occurrence of genital warts; recurrent respiratory papillomatosis; pre-cancerous lesions such as cervical intraepithelial neoplasia (CIN); cervical, vulvar, vaginal, penile, anal, and head/neck cancers related to these HPV genotypes. In the analyses presented here we focused only on the diseases included in the indication of the HPV quadrivalent vaccines, and therefore penile cancers, head and neck cancers and recurrent respiratory papillomatosis were excluded.

### 2.2 Input parameters

#### 2.2.1 Demographics and sexual behavior

The population of France and the annual all-cause mortality rates for the general population were retrieved from the French National Institute of Statistics and Economic Studies (*Institut National de la statistique et des études économiques,* INSEE) [24], [26], [27]. The French study published in 2008 was used for the sexual behavioral input for the model [28]. The amount of sexual mixing among members of different age cohorts and among members of different sexual activity groups were extracted from Supplementary tables 1–4 in a technical report from the United States [24], [25].

#### 2.2.2 Natural history of disease

The progression from infection to disease were set to follow a similar natural history structure to that in the initial US model [24]. As transmission rates are not directly observable, calibration techniques were used to obtain the best set of parameters.

#### 2.2.3 Cancer mortality

Survival data from the European Cancer Registry (EUROCARE-5) were used to estimate HPV-related cancer mortality. It was necessary to make the following assumptions: vaginal cancer mortality was assumed to be the same as that for vulvar cancer and mortality for anal cancer was assumed to be the same as that for both colon and rectum cancer. The five-year mortality rates were then converted to one-year death probabilities to provide the percentage of individuals with cancer expected to die in one year.

Since survival data were only available by age, we estimated the stage-stratified data using UK data from Cancer Research UK [29] to calculate the relative risk of each stage and applying this to the French specific survival statistics (assumed to be representative of regional stage) to calculate survival rates for local and distant cancers.

#### 2.2.4 Screening

The annual cancer screening rates and percentage of females undergoing cervical cancer screening at least every three years was extracted from a report from the French National Authority for Health (Haute Autorité de Santé; HAS) [5]. The percentage of females undergoing a repeat test after having an abnormal Pap smear test result was estimated to be 90.82% [30], [31]. The percentage of females undergoing regular vaginal cancer screening was set to 0% since no screening program for vulvar and vaginal cancer screening practice exists.

#### 2.2.5 HPV vaccines

Two HPV vaccines (Cervarix®, a bivalent vaccine immunizing against HPV 16/18 infections and Gardasil®, a quadrivalent vaccine immunizing against HPV 6/11/16/18 infections) are recommended in France. The quadrivalent vaccine occupies the majority of the French HPV vaccine market (around 85%) and as the model is unable to simulate mixed vaccination strategies, it was assumed that all vaccinees had received the quadrivalent vaccine.

The assumptions for the prophylactic efficacy of the vaccine were based on clinical trial data [32], [33], [34], [35], [36]. The duration of protection against HPV genotypes 6/11/16/18 was assumed to be lifelong, based on immunogenicity and effectiveness data for the quadrivalent HPV vaccine [37], [38] that have demonstrated efficacy lasting for up to ten years, and mathematical modeling of antibody decay following vaccination [39]. The model included estimates of protection against both infection and disease due to breakthrough infection, with different efficacy values for each. It was also assumed that these ‘breakthrough’ infections were transmissible. Efficacy against anal disease was assumed to be conferred through protection against infection only. Although the available vaccine efficacy data are not specific for the French population, it has been already shown that the US model can be transferable to other countries [40]. The vaccine efficacy parameters used in the model are summarized in Table 1.

**Table 1:** Summary of quadrivalent HPV vaccine efficacy assumptions.

Vaccine efficacy assumptions	HPV 16	HPV 18	HPV 6	HPV 11
Cervical cancer				

Prevention of cervical HPV infections:				
- Male^a^	0.411	0.621		
- Female^b^	0.760	0.963		
Prevention of HPV persistent infections	0.988	0.984		
Protection against HPV-related CIN	0.979	1		

Vaginal and vulvar cancers				

Prevention of VaIN/VIN infections:				
- Male^a^	0.411	0.621		
- Female^b^	0.760	0.963		
Prevention of persistent VaIN/VIN	0.988	0.984		
Protection against HPV-related VaIN/VIN	1	1		

Anal cancers				

Prevention of anal infections				
- Male^a^	0.411	0.621		
- Female^b^	0.760	0.963		

Protection against persistent anal infections				
- Male^a^	0.787	0.960		
- Female^b^	0.988	0.984		
Protection against HPV-related AIN neoplasia	0	0		

Genital warts				

Vaccine efficacy against HPV 6/11 infection				
- Female			0.761	0.761
- Males			0.490	0.570

Protection of the vaccine against HPV 6/11-related genital warts				
- Female			0.989	1
- Male			0.843	0.909
Protection against HPV 6/11-related CIN 1			1	1

AIN: Anal Intraepithelial Neoplasia; CIN: Cervical Intraepithelial Neoplasia; VaIN: Vaginal Intraepithelial Neoplasia; VIN: Vulvar Intraepithelial Neoplasia.

a Preventing male genital infections through male vaccination is assumed to prevent transmission of genital infections to females.

b Preventing female genital infections through vaccination is assumed to prevent transmission of genital infections to males.

#### 2.2.6 Time horizon

According to the model structure, the health outcomes were evaluated on a yearly basis. The assessment was conducted over a 100-year horizon because this was consistent with the time frame under which the system approached a steady state and the majority of the benefits of vaccination would be obtained [41].

### 2.3 Model calibration

The model was calibrated on incidence and mortality rates of HPV-related diseases observed in France. The calibration process involved many iterations to move the model outcomes closer to the targets. The following model outcomes were compared against the calibration target in each iteration: cervical cancer incidence, genital warts incidence, vaginal/vulvar/anal cancer incidence, and mortality rates for cervical/vaginal/vulvar cancer.

Most of the incidence data were found in a report developed by the World Health Organisation (WHO) and the Institut Català d'Oncologia (ICO) Information Centre on HPV and Cancer; the mortality data were collected from the French CépiDc (Centre d'épidémiologie sur les causes médicales de décès) database [42], [43], [44], [45]. The ICO Information Centre on HPV and Cancer compiles and centralizes updated worldwide data on HPV and HPV-related cancers. CépiDc is a database that presents annual national statistics on medical causes of death in France. We obtained incidence data for anal, vaginal and vulvar cancer from the ICO report [44]. The incidence and mortality data for cervical cancers were obtained from Binder-Foucard et al. [45]. Data on the incidence of CIN1, CIN2+ were obtained from Bergeron [31]. Data on the incidence of genital warts was obtained from Monsonego et al. [46].

The target incidences were calculated by multiplying the overall incidence and mortality rates by the proportions of diseases attributable to HPV infection found in the literature (Supplementary table 9) [3], [47], [48]. Target incidence and mortality rates are summarized in Table 2.

**Table 2:** Comparison of overall incidence between target and calibration.

	**Incidence -related to HPV 6/11/16/18**		**Mortality related to HPV 6/11/16/18**	
	**Target**	**Model output**	**Target**	**Model output**
**Female**				
Cervical cancer	6.78	6.75	2.51	2.48
CIN 2/3	102.90	22.49	–	–
Vaginal	0.28	0.07	0.29	0.02
Vulvar	0.12	0.10	0.64	0.03
Anal	1.27	1.29	0.55	0.40
Genital warts	194.57	195.36	–	–

**Male**				
Anal cancer	0.51	0.53	0.22	0.23
Genital warts	237.75	235.57	–	–

CIN: Cervical Intraepithelial Neoplasia.

### 2.4 Model analyses

**Fig. 1:**
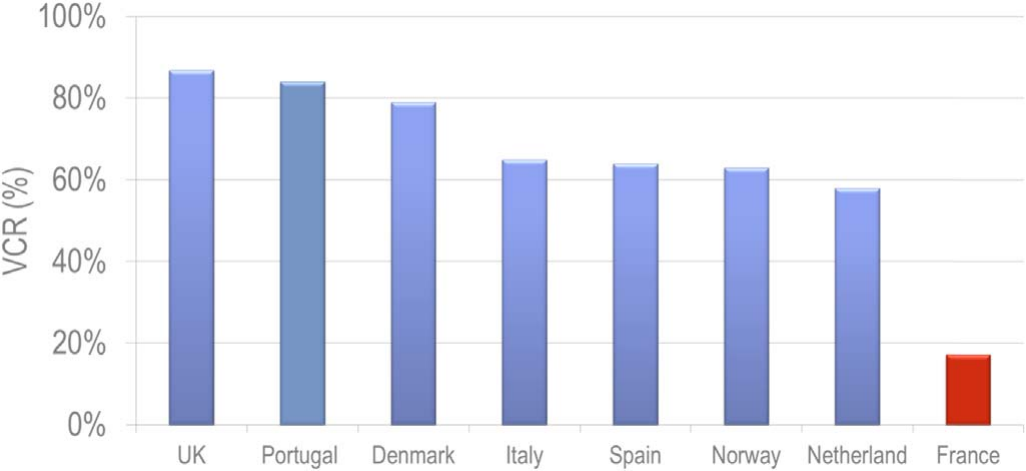
Vaccination coverage rates of HPV immunization program in EU countries in girls [14].

To quantify the impact of a low vaccination coverage rate (VCR), the health outcomes resulting from the vaccination of girls with quadrivalent HPV vaccine were assessed according to two theoretical vaccination coverage rates:

– a cumulative coverage rate of 17% at 16 years old corresponding to the vaccination coverage rate reported by the *Institut de Veille Sanitaire* in France in 2015.
– a cumulative coverage rate of 70% at 16 years old reflecting a level of protection frequently observed in the rest of Western Europe (Fig. 1) [14], [15], [49].

in addition, the two scenarios considered an increasing coverage rate, reaching the target vaccination coverage rate after 15 years.

As stated above, it is assumed in the analyses that all vaccinees receive the quadrivalent vaccine.

Scenarios with an alternative time horizon (70 years), vaccination coverage rate (60%), and vaccine duration of protection (20 years) were run in sensitivity analyses.

## 3 Results

### 3.1 Model calibration

The summary of the incidence targets collected from the literature and the model outputs are summarized in Table 2. After calibration, the model showed a good fit for the estimated incidence of cervical, anal and penile cancers, and genital warts. Of note, the results of the calibration showed that the incidence of CIN2/3 was underestimated by a factor 4.57 by the model.

### 3.2 Epidemiological results

Over 100 years, it was estimated that the 17% VCR observed in France would be associated with around more than 85,000 additional cancers, almost 28,000 additional deaths and more than 5 million diseases events overall compared with a 70% VCR (Table 3).

**Table 3:** Incremental number of additional disease events and deaths with a 17% vs 70% VCR.

	**Events**	**Deaths**
Cervical cancer	70,036	23,260
CIN2+	316,243	–
CIN1	181,638	–
Vaginal cancer	635	168
Vulvar cancer	910	269
Anal cancer females	10,421	2955
Anal cancer males	3053	1220
Genital Warts females	2,731,811	–
Genital Warts males	2,044,164	–
Total	5,358,911	27,872

CIN: Cervical Intraepithelial Neoplasia.

It was estimated that in the first ten years of the vaccination program, 73,837 additional diseases events including 73,067 genital warts, 346 CIN1, 421 CIN2+ and 3 cervical cancers would be averted with a 70% VCR scenario compared with 17% VCR.

**Fig. 2:**
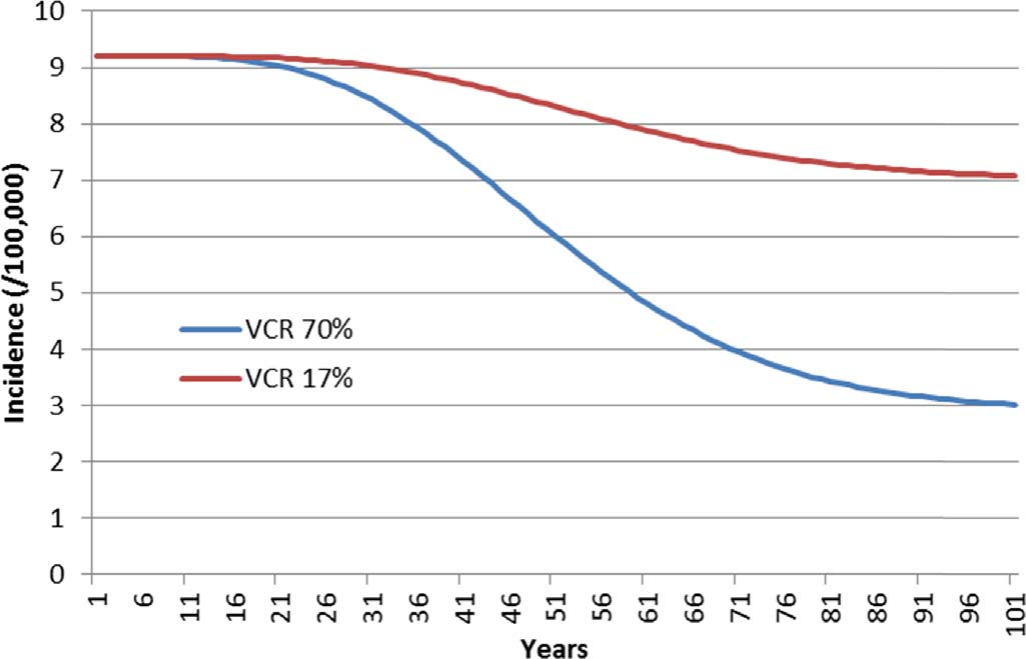
Incidence of cervical cancer over 100 years.

The model results showed that both strategies would have a significant impact on the epidemiology of cervical cancer, in particular, and HPV diseases, in general (Fig. 2; Table 4). At equilibrium (after 100 years), the annual number of cases was significantly lowered. It was estimated that about 1700 cancers, 600 deaths and 66,000 overall HPV-related disease events could be averted each year if the VCR in the French population was 70% rather than 17% (Table 5).

**Table 4:** Percentage decrease in HPV-related diseases incidence after 100 years.

	**VCR 70%**	**VCR 17%**
Cervical cancer	67	23
CIN2+	60	21
CIN1	22	8
Vaginal cancer	66	24
Vulvar cancer	68	25
Anal cancer females	72	23
Anal cancer males	50	13
Genital warts females	61	17
Genital warts males	37	9

CIN: Cervical Intraepithelial Neoplasia.

**Table 5:** Incremental number of events annually averted at equilibrium with a 70% VCR scenario vs a 17% VCR scenario.

	**Cases**	**Deaths**
Cervical cancer	1344	492
CIN2+	4528	–
CIN1	2532	–
Vaginal cancer	14	4
Vulvar cancer	20	6
Anal cancer females	242	75
Anal cancer males	79	34
Genital warts females	33,091	–
Genital warts males	24,258	–
**Total**	**66,108**	**611**

CIN: Cervical Intraepithelial Neoplasia.

### 3.3 Sensitivity analyses

According to the scenario, with a duration of vaccine protection of 20 years, the incremental number of events averted over 100 years ranged from more than 2,6 million cases to 5,3 million cases (base case) and the number of deaths averted ranged from more than 17,000 to almost 28,000 (base case) (Table 6).

**Table 6:** Incremental number of events averted according to the parameter tested in sensitivity analysis.

	**Cases**	**Deaths**
Base case	5,358,911	27,872
60% VCR	4,400,046	23,649
Time horizon=70 years	3,478,221	17,844
Duration of vaccine protection=20 years	2,683,981	17,293

VCR: Vaccination coverage rate.

## 4 Discussion

HPV vaccination is an essential component of HPV-related cancer prevention and control [50]. In the 2014 update of the European Code against cancer, HPV vaccination was included as one of the 12 key ways to reduce cancer risks with a recommendation to’ensure that children take part in vaccination programs for HPV’ [51]. HPV vaccination uptake is also considered as a key indicator for the quality of national cancer plans [52]. The successful implementation of HPV vaccination program as reflected by high vaccination uptake and equal access to vaccination is dependent on various factors such as the level of public awareness, parental vaccine acceptance, programmatic issues, healthcare professionals attitudes and importantly, political will [14].

There are several examples of unsuccessful vaccination programs. For example, the polio outbreak in Nigeria after suspicion that the polio vaccine was contaminated with antifertility drugs intended to sterilize young Muslim girls [53]. Another example is the confidence crisis in infant hepatitis B vaccination following notifications of post-vaccination neurological events in France [54]. Vaccine hesitancy, due to fear of side effects, is one of the key reasons for the poor uptake of HPV vaccination in France [55], 56]. Anti-vaccination activists communicate extensively about vaccine safety despite available pharmacovigilance data showing no vaccine safety concerns [57], while there is less communication about the benefits of HPV vaccination.

In the present modeling study, we estimated the consequences of sub-optimal HPV vaccination coverage, using the French context as an example. The results showed that if the vaccination coverage rate does not increase in France there could be a dramatic public health impact. Compared with the public health impact of a’standard’ vaccine coverage of 70%, the 17% coverage currently observed in France would be associated with a substantial loss of opportunity if this coverage rated were to remain constant. Over 100 years, this would correspond to more than 85,000 additional cancers, almost 28,000 additional deaths and more than 5 million avertable diseases events overall in the French population. Sensitivity analyses showed that the estimation of the number of events averted is sensitive to the assumptions for parameters whereas the estimation of the number of deaths averted is less sensitive. For example, under the assumption of a duration of vaccine protection of 20-year, the total number of case averted is halved while the total number of deaths averted is decreased by less than 40%. This is mainly explained by the impact on genital warts that have a high incidence and fast evolution compared with cancers.

The burden of HPV disease not averted will translate into an important economic burden through treatment and hospitalization costs, and sick leave or work days lost by a caregiver. Once the majority of the benefits of the vaccination program are obtained, i.e. when an epidemiologic equilibrium is reached, the annual burden would be strikingly different between the two coverage scenarios, since, at steady state, the current low vaccination coverage rate in France would be associated with an additional 1700 cancers, 600 deaths and 66,000 overall disease events each year as compared to the’standard’ EU vaccination coverage rate. If the HPV vaccine coverage rate of 60%, as targeted in the French Cancer Plan, were to be reached, most of this burden would be avoided [58]. However, some cohorts that have not been vaccinated, have already missed the opportunity to be protected. The model estimated that, in the first ten years of the program, the sub-optimal vaccination coverage rate would be associated with the occurrence of 73,067 genital warts, 346 CIN1, 421 CIN2+ and 3 cervical cancers, and this avertable burden will increase yearly as the unvaccinated cohorts become older.

Face validity of the model was demonstrated by several publications in peer-reviewed journals [23], [24]. Besides, the model was included in a meta-analysis conducted by Brisson et al. aiming to quantify the robustness of 16 HPV dynamic transmission models [62]. Notably, validity of one of the model was confirmed by reproducing was the decrease of genital warts incidence observed in real life in Australia. The authors concluded that “long-term population-level predictions were strikingly concordant, in particular in situations of high vaccination coverage among girls (>80%)”. In conclusion, even though limits remains regarding external validity, model structure used for the French analysis can be considered as acceptable and should be useful to illustrate the humanistic cost of a low vaccination coverage setting. The conclusions are thus likely to be generalizable to most high income countries presenting comparable VCR and vaccination strategies [62]. Real life data showing high effectiveness are now available in several settings that reached high HPV vaccination coverage. We could expect similar findings for the bivalent vaccine in terms of 16 and 18 related precancers and cancers that should be avoided if programs were fully implemented.

One of the potential limitations of the present study is that not all of the various model parameters were found in French-specific studies, which may have an impact on the validity of the results. Also, the CIN2+ incidence estimated by the model is 4.57 times lower than the observed CIN2+ incidence due to an imperfect calibration for this outcome. Hence, the “true” burden of CIN2+ associated with the low VCR is probably closer to 1,445,230 over 100 years than to the 316,243 cases estimated by the model. As a consequence, the burden associated with the low VCR was underestimated. Another potential limitation involves the assumptions of vaccination coverage rates that were based on 2014 French data. The VCR in the previous year and the distribution of the vaccinees among cohorts and by year were not taken into consideration, consequently, the estimations may not be fully representative of the French situation. This choice was made because of the lack of detailed data on the VCR. However, it is likely that the results and the conclusions would remain similar if the actual figures were used. French-specific sexual behavioral data were privileged whenever available [28]. However, one parameter “amount of sexual mixing” was not available, notably because of discrepancies between model structures. US-based original data was used [24]. Lastly, it was assumed that all model inputs would remain unchanged over the time horizon, which is a strong assumption since the value of the inputs would probable change, in particular, the vaccine used (a nonavalent HPV 6/11/16/18/31/33/45/52/58 vaccine, has been approved by the EMA in 2016), the vaccination coverage rate, screening practices and the disease burden. Despite these potential limitations, the study showed that the loss of chance due to the sub-optimal VCR would result in hundreds of deaths and thousands of disease events (cancers, precancers and genital warts) annually.

The importance of the missed opportunity is acknowledge by the French government which formally defined increasing HPV vaccination uptake as a public health priority in its Cancer Plan for 2014–2019 [63]. Two main objectives have been defined in the plan: (i) to reach a 60% VCR (ii) to assess HPV vaccination at school. Furthermore, several actions have been planned, such as the introduction of a pay-for-performance scheme related to HPV vaccination for physicians, diversification of the vaccination structures, including some providing the vaccine free of charge, authorization for nurses to administer the vaccine, increased communication on the benefit-risk profile of HPV vaccination, and promotion of social research on vaccination acceptability in school settings. These initiatives illustrate the necessity to act at multiple levels to improve HPV prevention.

A recent French survey showed that low educational and socioeconomic levels were associated with lower cervical cancer screening uptake in women and lower HPV vaccination rates in their daughters [64]. It is critical to improve the implementation of HPV vaccination strategies in France to reduce inequalities to access to cervical cancer prevention. A possible approach to improve HPV vaccination acceptance in France could be to lower the age of vaccination to 9 years, like it is now recommended in Germany, making it a routine childhood vaccination while offering optimal protection against HPV [65].

Vaccination of boys was not identified as a priority in France but this would constitute the most effective strategy to reduce the incidence of HPV-related cancers and diseases [14], [62]. Although HPV vaccination was originally indicated for the prevention of cervical cancer and targeted girls only, more and more EU countries now recommend and fund HPV vaccination programs for girls and boys (Austria, Switzerland, 9 regions in Italy) or are currently assessing the inclusion of boys in national programs (UK, Germany, Norway) [66], [67], [68], [69], [70]. Female and male vaccination programs appear to be the most effective strategy to break the chain of infection and prevent HPV-related morbidity and mortality in the population [14]. In France, a recommendation for vaccinating men having sex with men in specialized centers was issued in 2016 however the best way to protect all men is to vaccinate all males at a young age before potential exposure to the virus, irrespective of their sexual orientation [71]. In France, increasing the VCRs in girls is difficult since vaccination relies on self-referral. The inclusion of routine male vaccination could improve the success of the vaccination program by increasing VCR in the overall population which could contribute to maintaining high effectiveness of the vaccination program [72].

## 5 Conclusions

The low HPV vaccination coverage rate observed in France in 2014 highlights the difficulties this prevention program is facing. Timely, pertinent information on the burden of HPV and reassurance about the favorable benefit-risk ration are needed. Public health authorities and health care professionals have a key role to play in providing such information and strong recommendations to vaccinate to the public. Increasing the public trust in vaccination programs is essential if we are to achieve higher coverage with HPV vaccines. All stakeholders should work together and increase their efforts to achieve broader implementation of HPV vaccination and to enable HPV vaccination to deliver its full public health potential since partnerships could have positive outcomes on vaccination programs [6]. Effective communication on the importance of the loss of opportunity of not vaccinating the population is important because the avoidable disease burden can only be effectively avoided by high levels of VCR.

## Conflict of interest

M Adam, X Bresse, A Dahlab and M Uhart are employees of Sanofi Pasteur MSD, a pharmaceutical company that commercializes the HPV vaccines Gardasil and Gardasil9.

## Funding

This research did not receive any specific grant from funding agencies in the public, commercial, or not-for-profit sectors.

## References

[1] International Agency for Research on Cancer. IARC monographs on the evaluation of carcinogenic risks to humans. [100B]. 2012. PMC7681469 1683674

[2] Lacey C.J., Lowndes C.M., Shah K.V. (2006). Burden and management of non-cancerous HPV-related conditions: HPV-6/11 disease. Vaccine.

[3] Hartwig S., Baldauf J.J., Dominiak-Felden G. (2015). Estimation of the epidemiological burden of HPV-related anogenital cancers, precancerous lesions, and genital warts in women and men in Europe: potential additional benefit of a nine-valent second generation HPV vaccine compared to first generation HPV vaccines. Papillomavirus Res..

[4] Institut national du cancer. Cancer du col de l′uterus: quelques chiffres. 2016. 29-6-2016. 〈http://www.e-cancer.fr/Patients-et-proches/Les-cancers/Cancer-du-col-de-l-uterus/Quelques-chiffres〉 (Accessed 05 December 2016).

[5] Haute Autorité de Santé. Etat des lieux et recommandations pour le dépistage du cancer du col de l′utérus en France. 2010. 10.1016/j.jgyn.2011.02.004 21477950

[6] Garland S.M., Kjaer S.K., Munoz N. (2016). Impact and effectiveness Of the Quadrivalent human papillomavirus vaccine: a systematic review of Ten years of Real-world experience. Clin. Infect. Dis..

[7] Read T.R., Hocking J.S., Chen M.Y., Donovan B., Bradshaw C.S., Fairley C.K. (2011). The near disappearance of genital warts in young women 4 years after commencing a national human papillomavirus (HPV) vaccination programme. Sex. Transm. Infect..

[8] Chow E., Read T.R., Wigan R. (2014). Ongoing decline in genital warts among young heterosexuals 7 years after the Australian human papillomavirus (HPV) vaccination programme. Sex. Transm. Infect..

[9] Ali H., Donovan B., Wand H. (2013). Genital warts in young Australians five years into national human papillomavirus vaccination programme: national surveillance data. BMJ.

[10] Brotherton J.M., Saville A.M., May C.L., Chappell G., Gertig D.M. (2015). Human papillomavirus vaccination is changing the epidemiology of high-grade cervical lesions in Australia. Cancer Causes Control.

[11] Baandrup L., Blomberg M., Dehlendorff C., Sand C., Andersen K.K., Kjaer S.K. (2013). Significant Decrease in the incidence of genital warts in young danish women after implementation of a national human papillomavirus vaccination program. Sex Transm. Dis..

[12] Bollerup S., Baldur-Felskov B., Blomberg M., Baandrup L., Dehlendorff C., Kjaer S.K. (2016). Significant reduction in the incidence of genital warts in young men 5 years into the danish human papillomavirus vaccination program for girls and women. Sex. Transm. Dis..

[13] Herweijer E., Sundstrom K., Ploner A., Uhnoo I., Sparen P.+, Arnheim-Dahlstrom L. (2016). Quadrivalent HPV vaccine effectiveness against high-grade cervical lesions by age at vaccination: a population-based study. Int. J. Cancer.

[14] European Centre for Disease Prevention and Control. Introduction of HPV vaccines in EU countries - an update. 2012.

[15] Institut de Veille Sanitaire. Papillomavirus Humain - Données par groupe d′âge. 2016. 29-6-2016. 〈http://invs.santepubliquefrance.fr/Dossiers-thematiques/Maladies-infectieuses/Maladies-a-prevention-vaccinale/Couverture-vaccinale/Donnees/Papillomavirus-humains〉 (Accessed 05 May 2016).

[16] Butler R., MacDonald N.E. (2015). Diagnosing the determinants of vaccine hesitancy in specific subgroups: the Guide to Tailoring Immunization Programmes (TIP). Vaccine.

[17] Eskola J., Duclos P., Schuster M., MacDonald N.E. (2015). How to deal with vaccine hesitancy?. Vaccine.

[18] Salmon D.A., Dudley M.Z., Glanz J.M., Omer S.B. (2015). Vaccine hesitancy: causes, consequences, and a call to action. Vaccine.

[19] Forbes T.A., McMinn A., Crawford N., Leask J., Danchin M. (2015). Vaccination uptake by vaccine-hesitant parents attending a specialist immunization clinic in Australia. Hum. Vaccin. Immunother..

[20] Verger P., Fressard L., Collange F. (2015). Vaccine hesitancy among general practitioners and its determinants during controversies: a national cross-sectional survey in France. EBioMedicine.

[21] D. Lévy-Bruhl Estimation de l'impact épidémiologique des niveaux de couverture vaccinale insuffisants en France 2016. (http://www.academie-medecine.fr/publication100100485/) (Accessed 22 February 2017).

[22] E. Elbasha, E. Dasbach, R. Insinga, E. Elbasha, E. Dasbach, R. Insinga, Supplementary Online Appendix. 〈https://wwwnc.cdc.gov/eid/article/13/1/06-0438-techapp2.pdf〉, 2006.

[23] Elbasha E.H., Dasbach E.J., Insinga R.P. (2007). Model for assessing human papillomavirus vaccination strategies. Emerg. Infect. Dis..

[24] Elbasha E.H., Dasbach E.J. (2010). Impact of vaccinating boys and men against HPV in the United States. Vaccine.

[25] E.H. Elbasha, E.J. Dasbach, An integrated economic evaluation and HPV disease transmission models - Technical report accompanying the manuscrit "Impact on vaccinating boys and men against HPV in the United States", 2010. 10.1016/j.vaccine.2010.08.030 20713101

[26] Institut national de la statistique et des études économiques. Pyramide des âges au 1er janvier 2015. 2016. 29-6-2016. 〈https://www.insee.fr/fr/statistiques/1912926〉 (Accessed 05 December 2016).

[27] Institut national de la statistique et des études économiques. Décès, mortalité, espérance de vie. 2015. 29-6-2016. 〈https://www.insee.fr/fr/statistiques?Debut=0〉 (Accessed 05 December 2016).

[28] Bajos N., Bozon M., Beltzer N. (2008). Enquête sur la sexualité en France. Prat., genre Et. St., Paris, La Découverte.

[29] UK cancer research. Cervical cancer survival statistics. 2014.

[30] Bergeron C., Largeron N., McAllister R., Mathevet P., Remy V. (2008). Cost-effectiveness analysis of the introduction of a quadrivalent human papillomavirus vaccine in France. Int. J. Technol. Assess. Health Care.

[31] Bergeron C., Breugelmans J.G., Bouee S., Lorans C., Benard S., Remy V. (2006). Cervical cancer screening and associated treatment costs in France. Gynecol., Obstet. Fertil..

[32] Ault K.A. (2007). Effect of prophylactic human papillomavirus L1 virus-like-particle vaccine on risk of cervical intraepithelial neoplasia grade 2, grade 3, and adenocarcinoma in situ: a combined analysis of four randomised clinical trials. Lancet.

[33] Garland S.M., Hernandez-Avila M., Wheeler C.M. (2007). Quadrivalent vaccine against human papillomavirus to prevent anogenital diseases. N. Engl. J. Med..

[34] Giuliano A.R., Palefsky J.M., Goldstone S. (2011). Efficacy of quadrivalent HPV vaccine against HPV Infection and disease in males. N. Engl. J. Med..

[35] Joura E.A., Leodolter S., Hernandez-Avila M. (2007). Efficacy of a quadrivalent prophylactic human papillomavirus (types 6, 11, 16, and 18) L1 virus-like-particle vaccine against high-grade vulval and vaginal lesions: a combined analysis of three randomised clinical trials. Lancet.

[36] Palefsky J.M., Giuliano A.R., Goldstone S. (2011). HPV vaccine against anal HPV infection and anal intraepithelial neoplasia. N. Engl. J. Med..

[37] European Medicines Agency. Gardasil, summary of product characteristics. 2016.

[38] Nygard M., Saah A., Munk C. (2015). Evaluation of the long-term anti-human papillomavirus 6 (HPV6), 11, 16, and 18 immune responses generated by the quadrivalent HPV vaccine. Clin. Vaccin. Immunol..

[39] Fraser C., Tomassini J.E., Xi L. (2007). Modeling the long-term antibody response of a human papillomavirus (HPV) virus-like particle (VLP) type 16 prophylactic vaccine. Vaccine.

[40] Schobert D., Remy V., Schoeffski O. (2012). Cost-effectiveness of vaccination with a quadrivalent HPV vaccine in Germany using a dynamic transmission model. Health Econ. Rev..

[41] Ultsch B., Damm O., Beutels P. (2016). Methods for health economic evaluation of vaccines and immunization decision frameworks: a consensus framework from a European vaccine economics community. PharmacoEconomics.

[42] ICO information centre on HPV and cancer (HPV information centre). Human papillomavirus and related diseases in France: summary report. 2010.

[43] Centre d′épidémiologie sur les causes médicales de décès. Interrogation des données sur les causes de décès en 2011. 2011. 29-6-2016. 〈http://www.cepidc.inserm.fr/inserm/html/index2.htm〉 (Accessed 05 December 2016).

[44] ICO information centre on HPV and cancer (HPV information centre). Human papillomavirus and related diseases in Italy: summary report. 2014.

[45] Binder-Foucard F., Belot A., Delafosse P., Remontet L., Woronoff A.S., Bossard N. (2013). Estimation nationale de l′incidence et de la mortalité par cancer en France entre 1980 et 2012. Partie 1 - Tumeurs solides.

[46] Monsonego J., Breugelmans J.G., Bouee S., Lafuma A., Benard S., Remy V. (2007). Anogenital warts incidence, medical management and costs in women consulting gynaecologists in France. Gynecol., Obstet. Fertil..

[47] Hartwig S., Syrjoñnen S., Dominiak-Felden G., Brotons M., Castellsagué X. (2012). Estimation of the epidemiological burden of human papillomavirus-related cancers and non-malignant diseases in men in Europe: a review. BMC Cancer.

[48] J. Pretet, Human Papillomavirus (HPV) Genotype Distribution in Invasive Cervical cancers (ICC) in France: Results of the EDITH Study.: Idsa, 2006. 10.1002/ijc.23092 17893882

[49] Statens serum institut. Boernevaccinationsprogrammet. 2016. 29-6-2016. 〈http://www.ssi.dk/Vaccination/Boernevaccination/Boernevaccinationsprogrammet.aspx〉 (Accessed 05 December 2016).

[50] World Health Organization, Comprehensive Cervical Cancer Control: A Guide to Essential Practice. 〈http://apps.who.int/iris/bitstream/10665/144785/1/9789241548953_eng.pdf?Ua%3d1〉 (Accessed 10 September 2016), 2014. 25642554

[51] International Agency for Research on Cancer. European code against cancer. 2016. 29-9-2016. 〈http://cancer-code-europe.iarc.fr/index.php/en/〉 (Accessed 05 December 2016).

[52] European guide for quality national cancer control programme. Ljubljana: National Institute of Public Health, 2015.

[53] Minor P.D. (2004). Polio eradication, cessation of vaccination and re-emergence of disease. Nat. Rev. Microbiol..

[54] Denis F., Abitbol V. (2004). Aufr+¿re A. evolution des stratégies vaccinales et couverture vaccinale contre l′hépatite B en France, pays de faible endémie. M+®decine Et. Mal. Infect..

[55] Commission de la transparence. Gardasil: avis 20 mars 2013. 2016.

[bib56] Haut Conseil de la Santé Publique. Vaccination contre les infections à papillomavirus humains. 2014.

[57] Agence Nationale de Sécurité du Médicament et des produits de santé. Vaccination contre les infections à HPV et risque de maladies auto-immunes: une étude Cnamts/ANSM rassurante - point d′information. 2015. 29-6-2016. 〈http://ansm.sante.fr/S-informer/Points-d-information-Points-d-information/Vaccination-contre-les-infections-a-HPV-et-risque-de-maladies-auto-immunes-une-etude-Cnamts-ANSM-rassurante-Point-d-information〉 (Accessed 05 December 2016).

[58] Drolet M., Bénard E., Boily M.C. (2015). Population-level impact and herd effects following human papillomavirus vaccination programmes: a systematic review and meta-analysis. Lancet Infect. Dis..

[62] Brisson M., Bénard, Drolet M. (2016). Population-level impact, herd immunity, and elimination after human papillomavirus vaccination: a systematic review and meta-analysis of predictions from transmission-dynamic models. Lancet Public Health.

[63] Ministère des affaires sociales dlsedddf. Plan cancer 2014–2019. 2015.

[64] Guthmann J.P. (2012). Inégalités socioéconomiques d′accès à la vaccination contre les infections à papillomavirus humains en France. BEH.

[65] K. Robert, Institute. Epidemiologisches Bulletin Nr. 34. Report No.: 34, 2014.

[66] R. Wagner, Bundersministerium für gesundheit: Österreichischer impftag, 2015.

[67] Office Fédéral de la Santé Publique. Papillomavirus humains (HPV). 2015. 29-6-2016. 〈http://www.bag.admin.ch/themen/medizin/00682/00684/03853/index.html?Lang=fr〉 (Accessed 05 December 2016).

[68] The Vaccination Committee for the State of Saxony. Updated recommendations by the SIKO for vaccinations in the Free State of Saxony. 2013.

[69] Joint Committe on Vaccination and Immunisation. JCVI statement on HPV vaccination of men who have sex with men. 2015. 29-6-2016. 〈https://www.gov.uk/government/uploads/system/uploads/attachment_data/file/477954/JCVI_HPV.pdf〉 (Accessed 05 December 2016).

[70] Folkehelseinstituttet. Folkehelseinstituttet anbefaler HPV - vaksine til gutter. 2015. 29-6-2016. https://www.fhi.no/nyheter/2015/folkehelseinstituttet-anbefaler-hpv/ (Accessed 05 December 2016).

[71] Haut Conseil de la Santé Publique. Vaccination des garçons contre les infections à papillomavirus. 2016.

[72] Elfström K., Lazzarato F., Franceschi S., Dillner J., Baussano I. (2015). Human papillomavirus vaccination of boys and extended catch-up vaccination: effects on the resilience of programs. J. Infect. Dis..

