# Supplementary material for: Loss of chance associated with sub-optimal HPV vaccination coverage rate in France
Source: Papillomavirus Res. 2017 Feb 22;3:73–9. doi: 10.1016/j.pvr.2017.02.004 (PMC5883194; doi:10.1016/j.pvr.2017.02.004)
Supplement: Supplementary file 1 — Supplementary material [file mmc1.docx]

Supplementary materials.

Table 1. Percent of the population in each of the following sexual activity categories

| Category | Male (%) | Female (%) |
| --- | --- | --- |
| Low (mean number of sexual partners/year: ≤ 1) | 87.71 | 92.85 |
| Medium (mean number of sexual partners/year: 2-4) | 9.66 | 6.38 |
| High (mean number of sexual partners/year: 5+) | 2.63 | 0.77 |

Source: Bajos et al. (2008)

Table 2. Mean number of sexual partners per year by activity category and gender

| Sexual activity category | Males (number) | Females (number) |
| --- | --- | --- |
| Low (mean number of sexual partners per year: 0-1) | 0.86 | 0.84 |
| Medium (mean number of sexual partners per year: 2-4) | 2.65 | 2.58 |
| High (mean number of sexual partners per year: 5+) | 8.04 | 7.93 |

Source: Bajos et al. (2008)

The mean number of sexual partners per year by age group and gender was only available for the age groups between 18 and 69 in Bajos et al.. Thus, for the age group below 18, the number of sexual partners was estimated from the distribution observed in the US model from Elbasha. For the age groups above 70 years, the mean number of sexual partners was estimated from the previous age group: two third of the 65-69 mean number of sexual partners.

Table 3. Mean number of sexual partners per year by age group and gender

| Age Group | Male | Female |
| --- | --- | --- |
| 13-14* | 0.050 | 0.078 |
| 15-17* | 0.624 | 0.972 |
| 18-19 | 1.3 | 1.2 |
| 20-24 | 1.5 | 1.2 |
| 25-29 | 1.4 | 1.2 |
| 30-34 | 1.4 | 1.1 |
| 35-39 | 1.2 | 1.1 |
| 40-44 | 1.1 | 1.0 |
| 45-49 | 1.3 | 1.0 |
| 50-54 | 1.2 | 0.9 |
| 55-59 | 1.2 | 0.8 |
| 60-64 | 0.9 | 0.8 |
| 65-69 | 0.9 | 0.6 |
| 70+ | 0.6 | 0.4 |

Source: Bajos et al. (2008)

* Assumption based on Elbasha et al. (2010)

The amount of sexual mixing among members of different age cohorts (a value between 0 and 1 with 0 representing no mixing, and 1 representing maximum mixing) and the amount of sexual mixing among members of different sexual activity groups required in the model were extracted from the technical report accompanying the Manuscript “Impact of Vaccinating Boys and Men against HPV in the United States". Sexual mixing values are reported in Table 4.

Table 4. Sexual mixing

| Ages | Sexual mixing |
| --- | --- |
| **Among members of different age cohort** |  |
| Between debut and cessation | 0.40 |
| After cessation | 0.10 |
| **Among members of different sexual activity groups** | 0.50 |

Source: Elbasha et al. (2010)

Table 5: Annual cancer-associated mortality by site, age and stage

| Cancer type | Age group (years) | Mortality rate | | |
| --- | --- | --- | --- | --- |
|  |  | **Local Cervical Cancer** | **Regional Cervical Cancer** | **Distant Cervical Cancer** |
| **Cervical cancer** | 0-14 | 0.000 | 0.000 | 0.000 |
|  | 15-44 | 0.016 | 0.052 | 0.132 |
|  | 45-54 | 0.023 | 0.074 | 0.187 |
|  | 55-64 | 0.035 | 0.112 | 0.282 |
|  | 65-74 | 0.038 | 0.119 | 0.300 |
|  | >75 | 0.072 | 0.229 | 0.578 |
| **Vaginal cancer** | 0-14 | 0 | 0 | 0 |
|  | 15-44 | 0.030 | 0.051 | 0.092 |
|  | 45-54 | 0.049 | 0.085 | 0.151 |
|  | 55-64 | 0.065 | 0.113 | 0.201 |
|  | 65-74 | 0.079 | 0.137 | 0.244 |
|  | >75 | 0.140 | 0.242 | 0.430 |
| **Vulvar cancer** | 0-14 | 0 | 0 | 0 |
|  | 15-44 | 0.024 | 0.051 | 0.111 |
|  | 45-54 | 0.039 | 0.085 | 0.183 |
|  | 55-64 | 0.052 | 0.113 | 0.244 |
|  | 65-74 | 0.063 | 0.137 | 0.295 |
|  | >75 | 0.111 | 0.242 | 0.522 |
| **Anal cancer (Females)** | 0-14 | 0 | 0 | 0 |
|  | 15-44 | 0.027 | 0.061 | 0.104 |
|  | 45-54 | 0.033 | 0.074 | 0.127 |
|  | 55-64 | 0.034 | 0.076 | 0.130 |
|  | 65-74 | 0.049 | 0.109 | 0.187 |
|  | >75 | 0.086 | 0.192 | 0.330 |
| **Anal cancer (Males)** | 0-14 | 0 | 0 | 0 |
|  | 15-44 | 0.035 | 0.078 | 0.135 |
|  | 45-54 | 0.041 | 0.091 | 0.157 |
|  | 55-64 | 0.045 | 0.100 | 0.172 |
|  | 65-74 | 0.058 | 0.129 | 0.222 |
|  | >75 | 0.100 | 0.222 | 0.382 |

Source: Eurocare 5 and Cancer research UK

Table 6. Percentage of females screened for cervical cancer in the past year in France

| Age group (years) | Value Females (percent) |
| --- | --- |
| 1-19 | 0.00 |
| 20-24 | 18.42 |
| 25-29 | 25.71 |
| 30-34 | 27.25 |
| 35-39 | 27.44 |
| 40-44 | 26.88 |
| 45-49 | 25.47 |
| 50-54 | 21.92 |
| 55-59 | 19.23 |
| 60-64 | 16.56 |
| 65-69 | 13.33 |
| >70 | 0.00 |

Source: Based on HAS (2010)

Table 7. Probability of transmitting genital HPV infection per sexual partnership, by sex and HPV genotype

| Transmission | HPV 16 | HPV 18 | HPV 6 | HPV 11 | HPV type 31, 33, 45, 52 or 58 |
| --- | --- | --- | --- | --- | --- |
| To males | 0.1109 | 0.1109 | 0.2577 | 0.2577 | 0.076 |
| To females | 0.1109 | 0.1109 | 0.2577 | 0.2577 | 0.076 |

Source: Hernadez and calibration process

Table 8. Parameters of natural history of disease

| Parameters (References) | HPV 16 | HPV 18 |
| --- | --- | --- |
| Fraction of persistent cervical HPV infections | 0.25 | 0.075 |
| Clearance rate of cervical HPV infections |  |  |
| Male | 0.39550 | 0.37755 |
| Female | 0.354 | 0.348 |
| Fraction of people seroconvert following a cervical HPV infection |  |  |
| Male | 0.6 | 0.6 |
| Female | 0.6 | 0.6 |
| Degree of protection against cervical HPV infections provided by natural immunity following seroconversion |  |  |
| Male | 0.5 | 0.5 |
| Female | 0.8 | 0.8 |
| Fraction of females transiently infected with HPV16 progress to CIN over the course of one year |  |  |
| CIN 1 | 0.105 | 0.068 |
| CIN 2 | 0.045 | 0.055 |
| CIN 3 | 0.024 | 0.009 |
| Probability of transmitting anal HPV infection (Calibration) |  |  |
| To males | 0.16 | 0.16 |
| To females | 0.173 | 0.173 |
| Probability of transmitting penile HPV infection (Calibration) |  |  |
| To males | 0.123 | 0.123 |
| To females | 0.123 | 0.123 |
| Probability of transmitting head and neck HPV infection (Calibration) |  |  |
| To males | 0.14118 | 0.13228 |
| To females | 0.14118 | 0.13228 |

Source: Elbasha, Ho, Onda, Insinga and calibration

Table 9. Proportion of HPV-related cancers and HPV-related diseases

|  | Prevalence  of cancers or GW due to HPV | | Prevalence of HP16/18 in HPV+ cancers or precancerous lesions | | Prevalence of HPV 6/11 in HPV+ GW | HPV 16/18/6/11 | |
| --- | --- | --- | --- | --- | --- | --- | --- |
|  |  |  |  | |  | **Female** | **Male** |
| **Cervical precancerous lesions** | |  | |  |  |  |  |
| CIN 1 | 100% | | 24.0% | |  | 24.0% | - |
| CIN 2+ | 100% | | 64.0% | |  | 64.0% | - |
| **Cancers** |  | |  | |  |  |  |
| Cervical | 100% | | 73.7% | |  | 73.7% | - |
| Vaginal | 71.1% | | 71.3% | |  | 50.7% | - |
| Vulvar | 19.3% | | 73.5% | |  | 14.2% | - |
| Anal | 87.6% | | 87.1% | |  | 76.3% | 76.3% |
| **Genital warts** | 100% | |  | | 85.0% | 85.0% | 85.0% |

GW: Genital Warts; CIN: Cervical Intraepithelial Neoplasia;

Source: Hartwig et al. (2012), Hartwig et al. (2015) Pretet et al. (2006)

Figure 1: Age-specific calibration of HPV 6/11/16/18 cervical cancer incidence


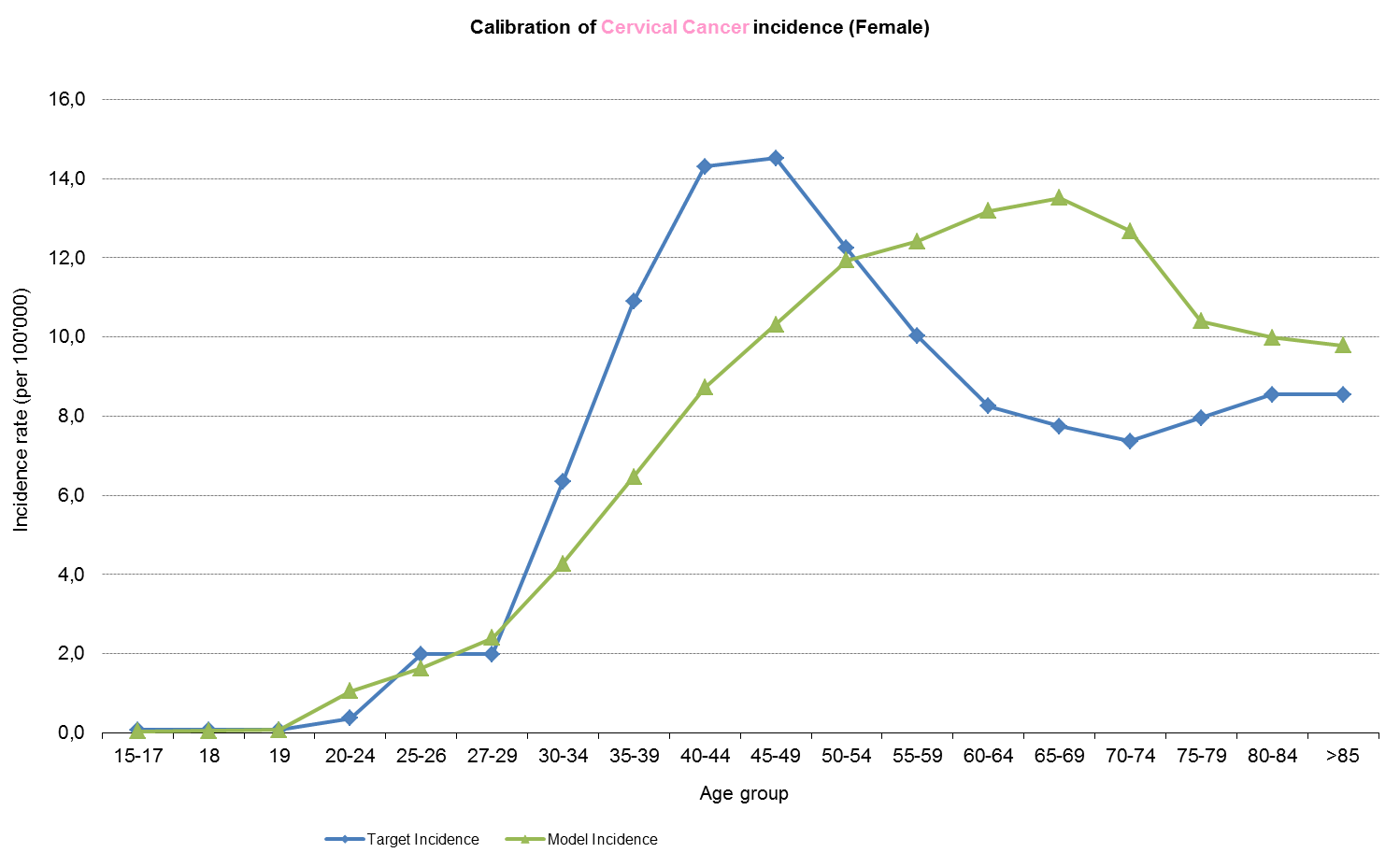


Figure 2: Age-specific calibration of HPV 6/11/16/18 CIN 2+ incidence


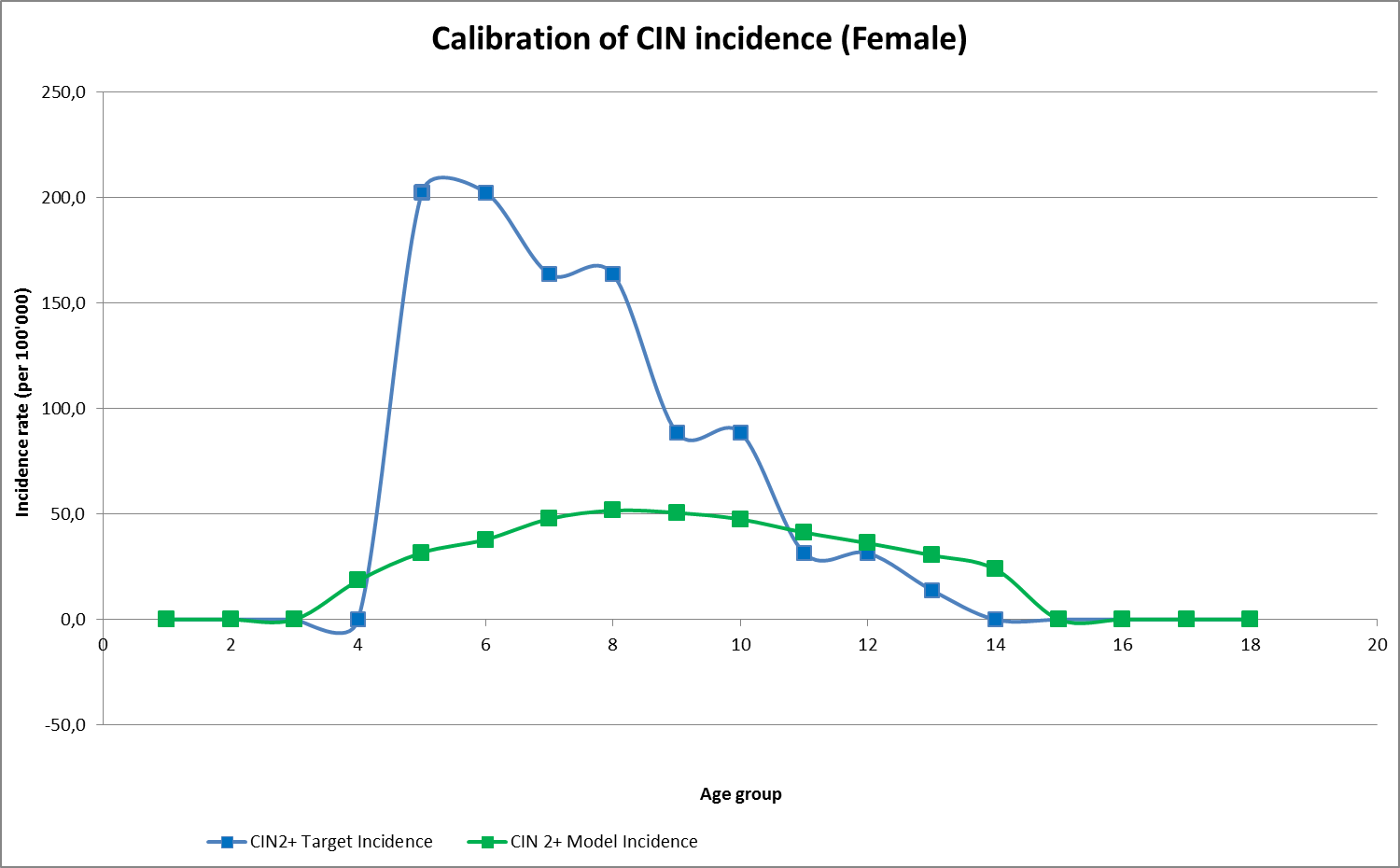


Figure 3: Age-specific calibration of HPV 6/11 genital warts incidence in females


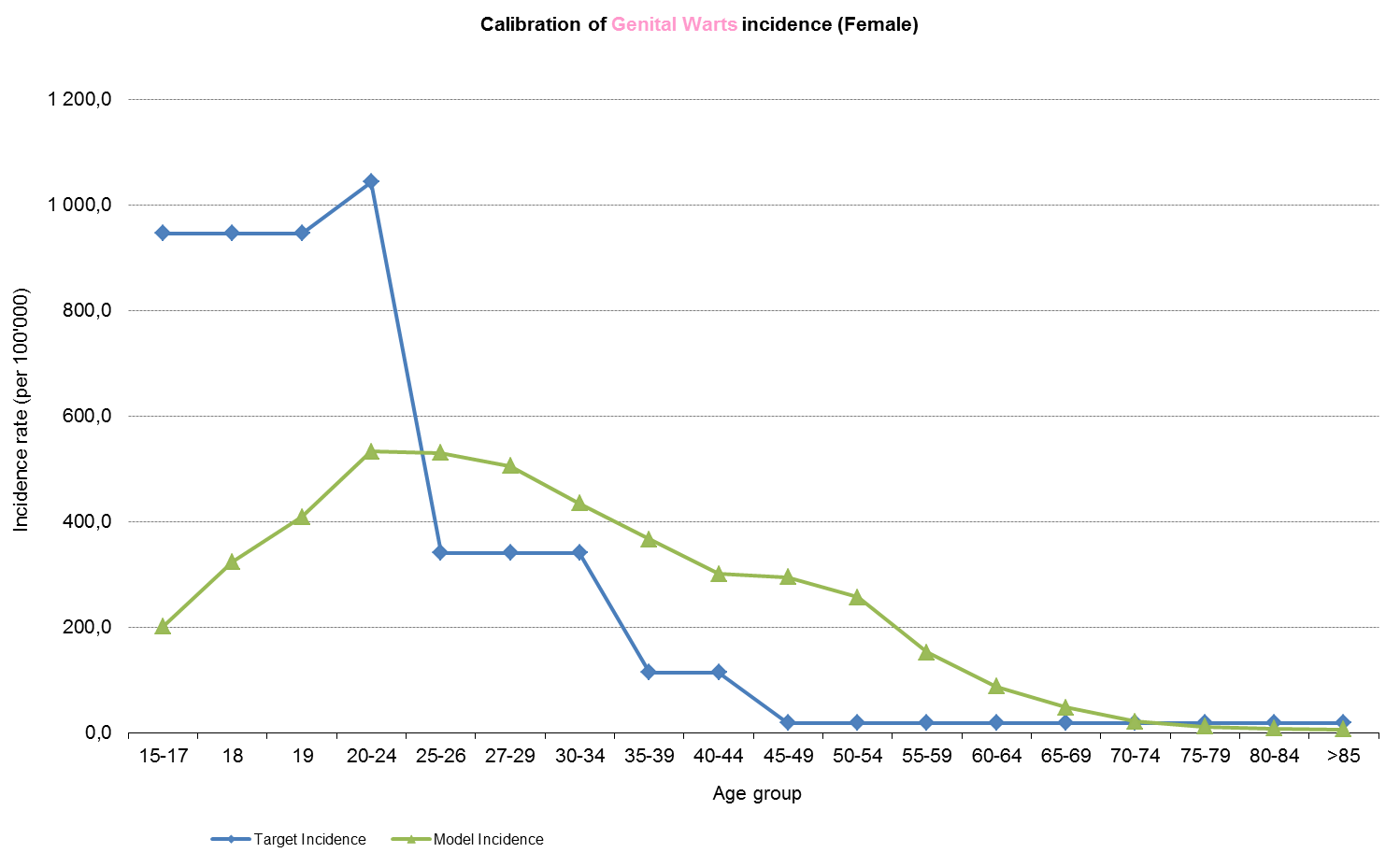


Figure 4: Age-specific calibration of HPV 6/11 genital warts incidence in males


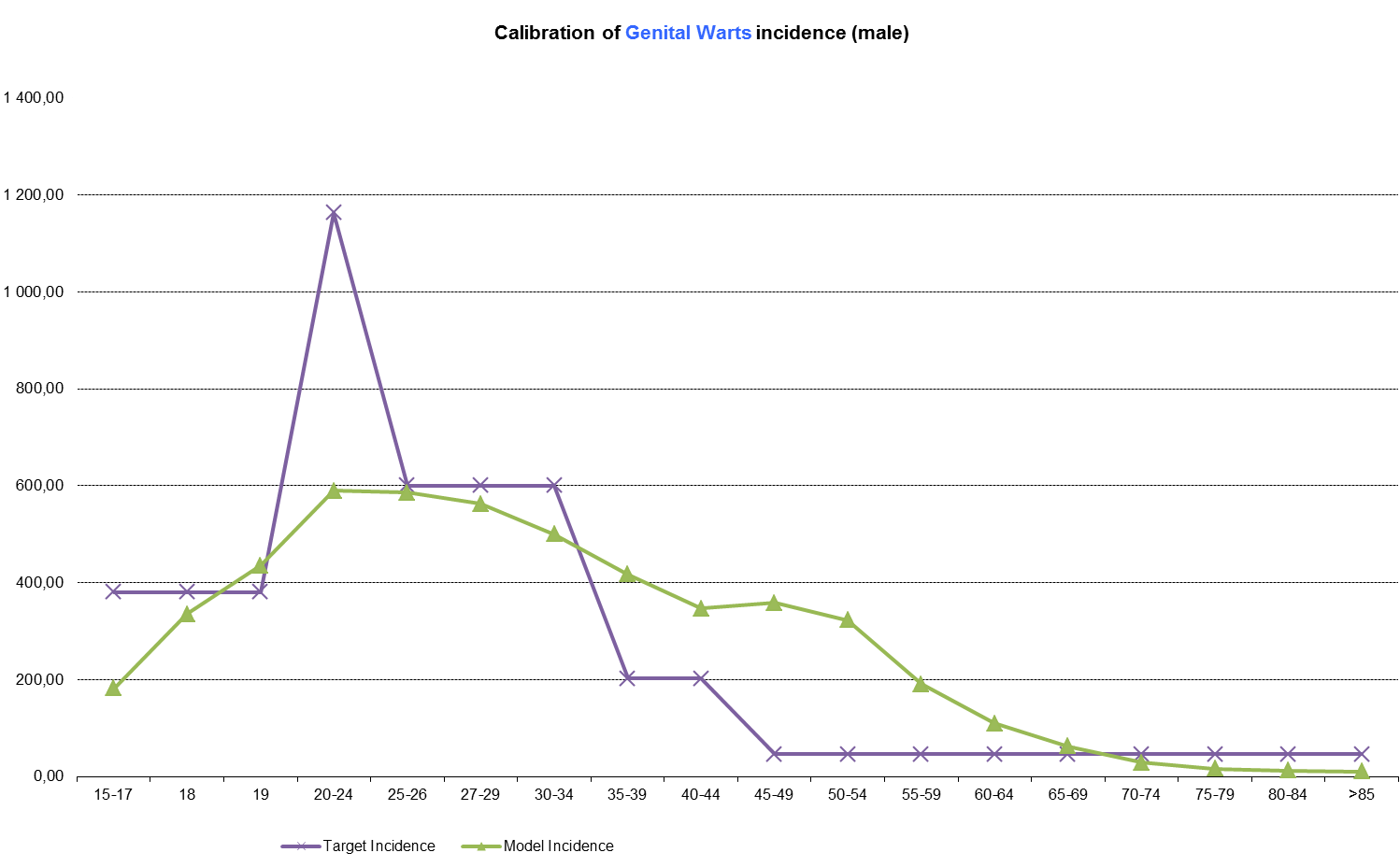


Figure 5: Age-specific calibration of HPV 6/11/16/18 vaginal cancer incidence


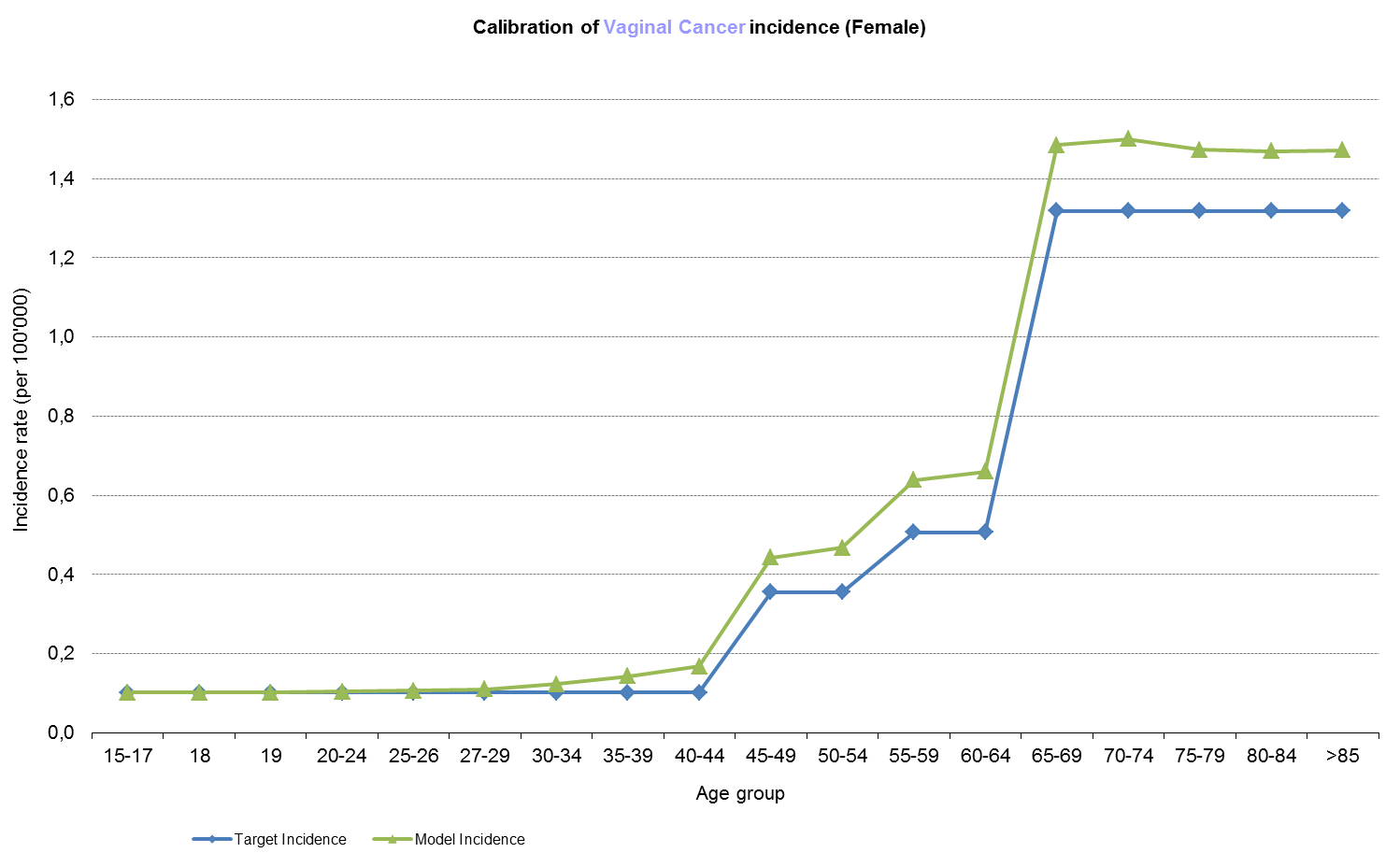


Figure 6: Age-specific calibration of HPV 6/11/16/18 vulvar cancer incidence


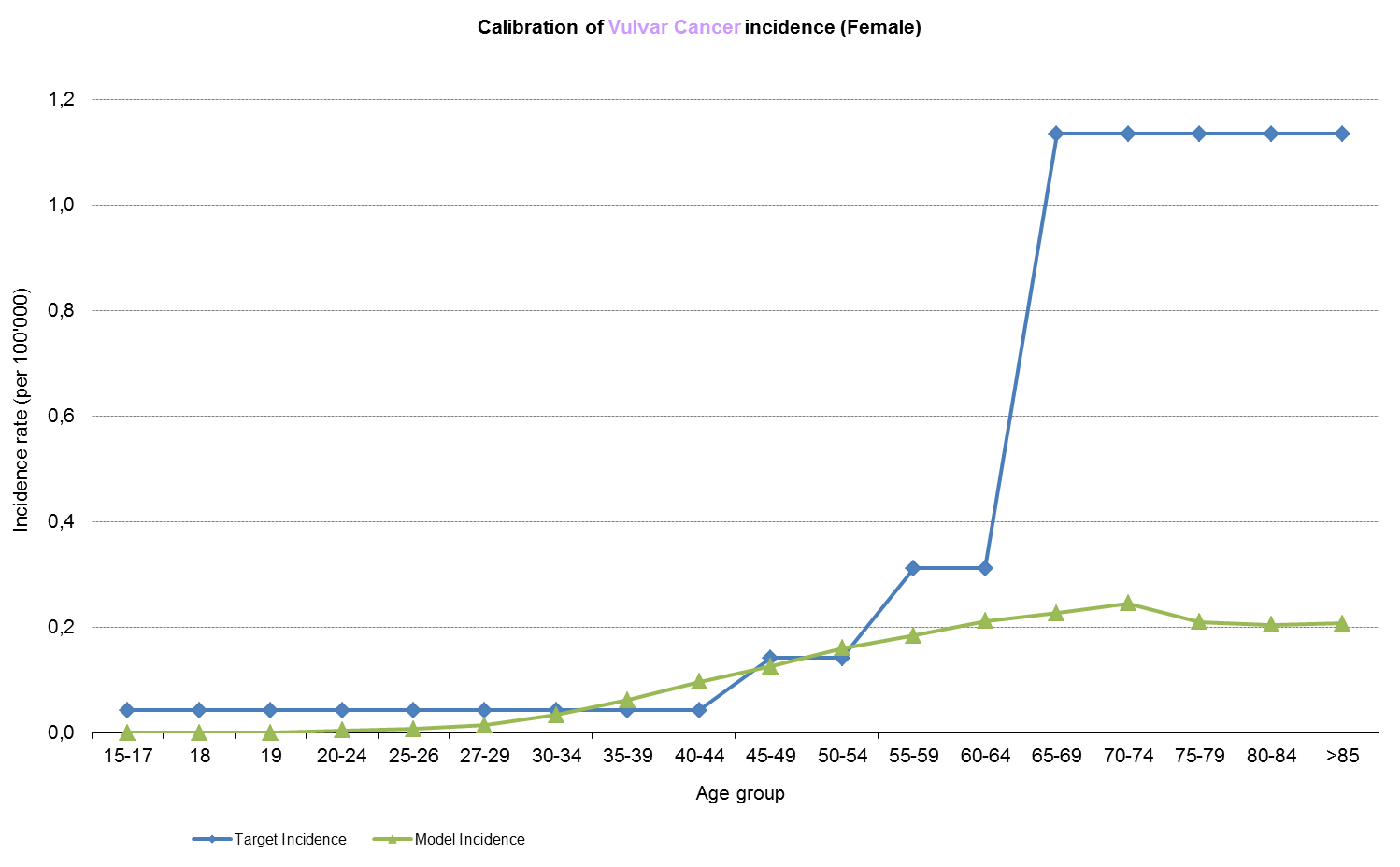


Figure 7: Age-specific calibration of HPV 6/11/16/18 anal cancer incidence in females


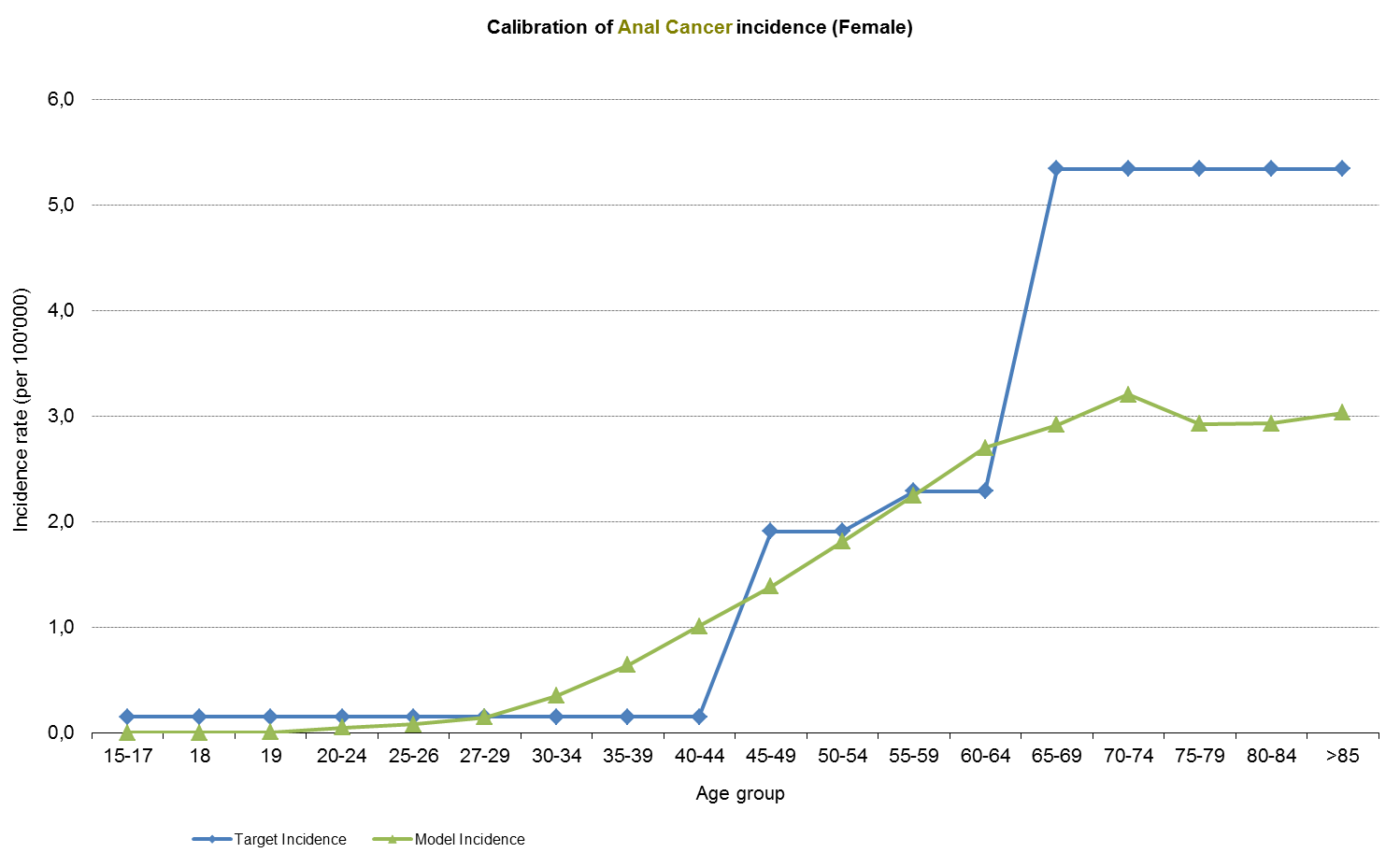


Figure 8: Age-specific calibration of HPV 6/11/16/18 anal cancer incidence in males


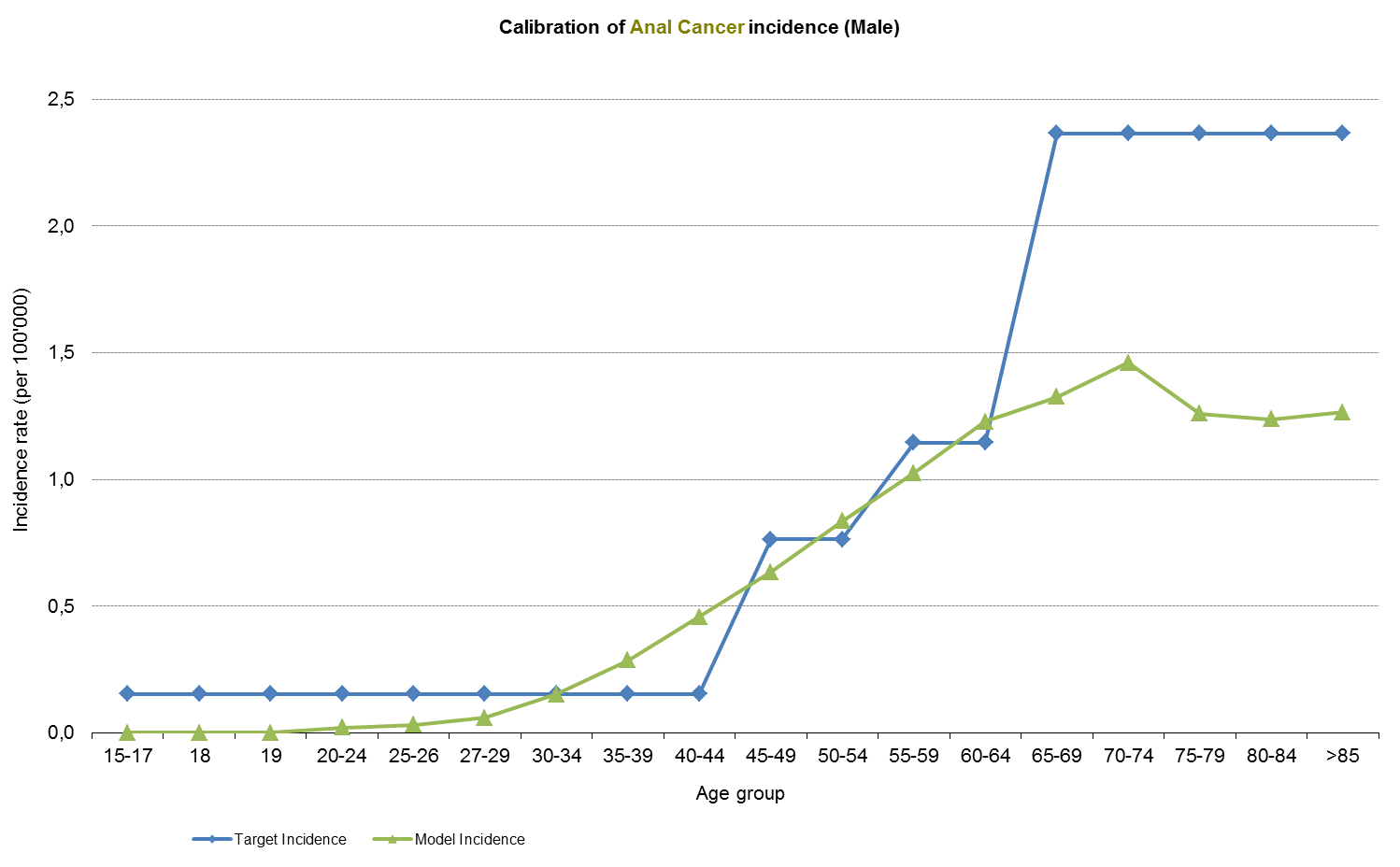


**References:**

Bajos, N. and M. Bozon, *Enquête sur la sexualité en France. Pratiques, genre et santé*. 2008, **Paris**.

*Cancer statistics*. 2015 7/5/2015; Available from: <http://www.cancerresearchuk.org/cancer-info/cancerstats/types/>.

Elbasha, E.H. and E.J. Dasbach, *An Integrated Economic evaluation and HPV disease transmission models - Technical report accompanying the Manuscript "Impact of vaccinating Boys and Men against HPV in the Inited States.* Vaccine, 2010. **28**(42): p. 6858-6867.

*Eurocare Survival of cancer patient in Europe*. 2015, Istituto Nazionale Tumori Istituto Superiore di Sanità.

Hartwig, S., et al., *Estimation of the epidemiological burden of human papillomavirus-related cancers and non-malignant diseases in men in Europe: a review.* BMC.Cancer, 2012. **12**: p. 30.

*État des lieux et recommandations pour le dépistage du cancer du col de l'utérus en France*. 2010, Haute Autorité de Santé.

Hartwig, S., J.J. Baldauf, and G. Dominiak-Felden, *Estimation of the epidemiological burden of HPV-related cancers, precancerous lesions, and genital warts in women and men in Europe: potential additional benefit of a nine-valent compared to the quadrivalent HPV vaccine*. 2015.

Hernandez, B.Y., et al., *Transmission of human papillomavirus in heterosexual couples.* Emerg.Infect.Dis., 2008. **14**(6): p. 888-894.

Ho, G.Y., et al., *Natural history of human papillomavirus type 16 virus-like particle antibodies in young women.* Cancer Epidemiol.Biomarkers Prev., 2004. **13**(1): p. 110-116.

Insinga, R.P., et al., *Progression and regression of incident cervical HPV 6, 11, 16 and 18 infections in young women.* Infect.Agent.Cancer, 2007. **2**: p. 15.

Onda, T., et al., *Characterization of IgA response among women with incident HPV 16 infection.* Virology, 2003. **312**(1): p. 213-221.

Pretet, J.L., A.C. Jacquard, and X. Carcopino, *Human papillomavirus (HPV) Genotype Distribution in Invasive Cervical cancer (ICC) in France: Results of the EDITH study*, in *Annual Meeting of the Infectious Diseases Society of America*. 2006: Toronto.
